# Associations of lactate dehydrogenase with risk of respiratory disease mortality among cancer survivors

**DOI:** 10.1097/MD.0000000000047466

**Published:** 2026-02-06

**Authors:** Liangjun Tang, Jide Chen, Haibo Tan

**Affiliations:** a Department of Clinical Laboratory, Bishan Hospital of Chongqing Medical University, Chongqing, China.

**Keywords:** cancer, lactate dehydrogenase, NHANES, respiratory disease mortality

## Abstract

Respiratory disease is a leading cause of death among cancer survivors. Using NHANES 2007–2012 data (15,854 participants, 1472 with cancer), we examined whether the lactate-dehydrogenase-to-albumin ratio (LAR) predicts respiratory mortality. LAR was calculated as LDH (U/L) divided by serum albumin (g/L), and participants were categorized into quintiles. The study found that higher LAR levels were positively correlated with increased all-cause and respiratory disease mortality. Specifically, participants in the highest LAR quintile had a 43% higher risk of all-cause mortality and a significantly higher risk of respiratory disease mortality (hazard ratio = 4.87, 95% confidence interval: 1.25–18.9). These data indicate that LAR may serve as an easily available prognostic marker to flag cancer survivors at high risk of fatal respiratory events, informing targeted surveillance and early intervention.

## 1. Introduction

Respiratory disease is one of the most common causes for mortality in patients with cancers.^[1]^ These complications include infections such as pneumonia, which can cause symptoms like fever, cough, and shortness of breath, severely affecting patients’ daily activities and mental health.^[2]^ Pulmonary embolism is another serious complication, leading to sudden shortness of breath, chest pain, and even hemoptysis, posing a life-threatening risk. Respiratory failure, often occurring in advanced cancer patients, results in severe hypoxia and hypercapnia, further worsening the patients’ prognosis.^[3]^ These respiratory complications not only reduce the patients’ physical quality of life but also cause significant psychological stress, leading to decreased overall quality of life. More importantly, due to the outbreak of Coronavirus Disease 2019 (COVID-19) and compromised immune systems, the rate of respiratory mortality in these patients was significantly increased.^[4]^ Therefore, it is of great importance to screen for a group of patients with higher risk for respiratory disease among cancer survivor.

The lactate dehydrogenase to serum albumin ratio (LAR) has been widely studied in various researches and has shown potential applications for the diagnosis and prognosis among various respiratory diseases and malignancies. For instance, increased LAR is associated with poor prognosis in patients with lower respiratory tract infection patients and pulmonary embolism and pulmonary infection.^[5,6]^ Moreover, LAR could also be utilized for predicting survival in malignancies including colorectal cancer, bladder Cancer and gastric cancer.^[7-9]^ However, the exact association between LAR and respiratory-related mortality in cancer patients has not been elucidated. In this study, we found that LAR was positively correlated with the higher all-death cause and respiratory disease mortality among patients. This study highlights LAR’s role in predicting mortality risks in cancer patients, offering vital insights for prognosis.

## 2. Methods and materials

### 2.1. Study population

**Figure 1.**
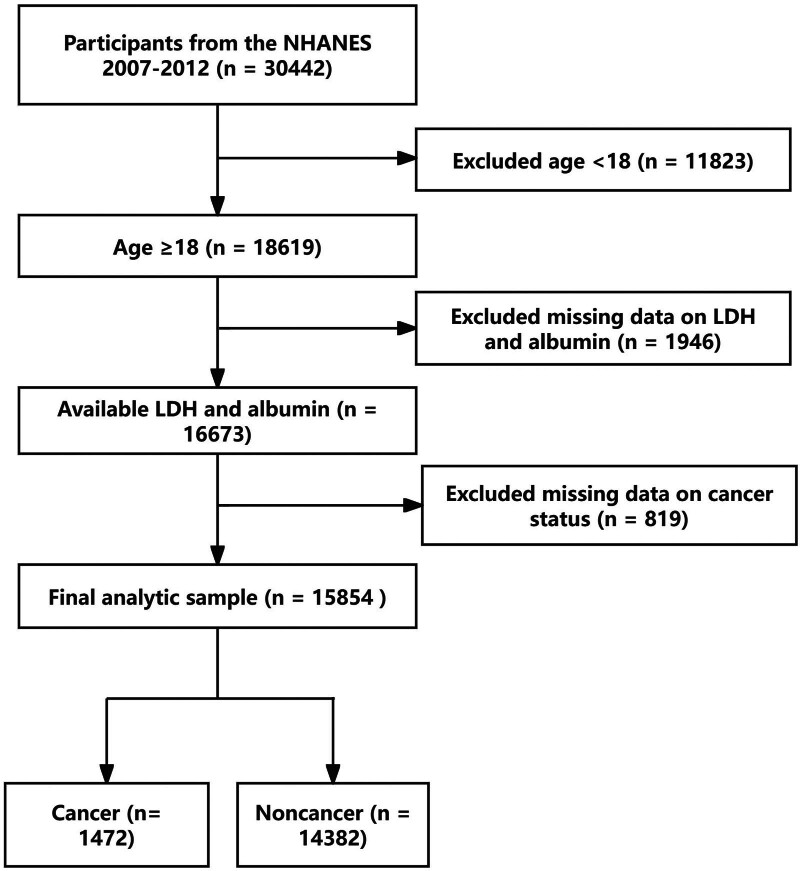
Workflow of the research. A total of 1472 cancer patients and 14,382 non-cancer patients are included in the final analysis. LDH = lactate dehydrogenase, NHANES = National Health and Nutrition Examination Survey.

All the data are from the US NHANES database. The NHANES, an epidemiological initiative spearheaded by the National Center for Health Statistics (NCHS) under the Centers for Disease Control and Prevention, aims to evaluate the health and nutritional status of the noninstitutionalized civilian resident population in the United States. Commencing in 1999, the sampling design has incorporated a multiyear, stratified, clustered 4-stage approach, with data being disseminated in 2-year cycles. In this study, we initially included 30,442 participants who participated in the NHANES (2007–2012) survey and then the participants under the age of 18 were removed. Next, we also excluded a total of 1946 participants who lacked LDH and albumin data and a total of 819 patients lacking data of cancer status. Finally, a large number of 15,854 were included in the data statistics (Fig. 1). The study protocol of NHANES had been granted approval by the Ethics Review Board of the National Center for Health Statistics and Research. All participants had provided written informed consent, authorizing the utilization of their data. All experiments presented in this paper were conducted in strict accordance with relevant guidelines and regulations.^[10]^

### 2.2. Definitions

LAR was computed as LDH (U/L)/serum albumin (g/L).^[11]^ Patients were categorized into 5 subgroups (Q1, Q2, Q3, Q4, and Q5) based on LAR levels, which were respectively 0 to 2.681, 2.681 to 3.026, 3.026 to 3.358, 3.358 to 3.828, and 3.828 to 9.533.

### 2.3. Covariates

Covariates were chosen through a directed review of the literature.^[12]^ A standardized questionnaire was employed to collect sociodemographic information, including age, sex, race/ethnicity, family poverty income ratio, educational attainment, marital status, body mass index (BMI), smoking status. Participants self-reported their race and ethnicity according to the categories defined by the NCHS, which include Mexican American, other Hispanic, non-Hispanic White, non-Hispanic Black, and other races/ethnicities (such as American Indian/Alaskan Native/Pacific Islander, Asian, and multiracial). The family income-to-poverty ratio was divided into 3 groups: ≤1.30, 1.31 to 3.50, and >3.50.^[13]^ Educational level was categorized as “less than high school diploma,” “high school diploma or general equivalency diploma,” or “some college or above.” Marital status was classified into 4 categories: married, never married, living with a partner, and other (including widowed, divorced, or separated). BMI was calculated by weight in kilograms/(height in meters).^[2]^ Participants were categorized as never, current, or former smokers based on self-reported smoking history (lifetime cigarette count < 100, ≥100 and currently smoking, or ≥100 and quit).^[14]^

### 2.4. Mortality ascertainment

The study’s primary endpoints were all-cause and respiratory disease mortality, ascertained via publicly available mortality data from the NCHS, with follow-up through December 31, 2019. Causes of death were coded according to the ICD-10. All-cause deaths were defined as mortality from any cause and deaths caused by respiratory disease were identified using ICD-10 codes J09-J18 and J40-J47.^[12,14]^ The follow-up duration was calculated from the interview date to the date of death or to December 31, 2019, for those without an event. Cancer status was tracked via the NHANES-NDI file to 31 December 2019 (probabilistic linkage) literature.^[12]^

### 2.5. Statistical analysis

According to the recommendation from NHANES analytic guidelines, the sampling design and weights were considered for specific groups to ensure a representative result.^[10]^ Categorical variables were presented as percentages and analyzed via χ^2^ or Fisher’s exact tests. Continuous data were compared using *t*-tests or Wilcoxon rank sum test due to data distribution.

Cox proportional hazards regression models were utilized to estimate hazard ratios (HRs) and 95% confidence intervals (CIs) for the relationships between LAR levels and all-cause, respiratory disease mortality, with adjustments made for covariates other than the variables themselves. Participants were grouped by LAR levels to assess mortality risks and explore joint associations, using multivariable Cox proportional hazards regression models with the same set of covariates. A subgroup analysis was furtherly performed to assess the associations between LAR quartiles (Q1–Q5) and all-cause and respiratory mortality. Analyses were conducted in R 4.3.0.

## 3. Results

### 3.1. Baseline characters

The study population was derived from the NHANES 2007–2012 dataset, with the final analytic sample including 15,854 participants, of whom 1472 had a diagnosis of cancer. The selection process is illustrated in Figure 1, with exclusions based on age, missing data on lactate dehydrogenase (LDH) and albumin, and missing data on cancer status. The baseline characteristics and weighted estimates of the study population are presented in Table 1, with a total of 1472 participants categorized into quintiles based on the lactate dehydrogenase to LAR. The mean age was 59.6 years (95% CI: 58.5–60.7), and the population was predominantly female (56.3%) and non-Hispanic White (87.3%). The family poverty income ratio showed 46.1% of participants had a ratio > 3.5, and 61.9% had a college education or higher. Marital status indicated 63.8% were married. The mean BMI was 28.1 kg/m^2^ (95% CI: 27.7–28.5), with 47.0% of participants being never smokers.

**Table 1:** Baseline characteristics of 1472 participants according to LAR from NHANES 2007–2012.

Characteristics	Number of weighted %*					
	Total (n = 1472)	LAR				
		Q1 (0–2.681) (n = 295)	Q2 (2.681–3.026) (n = 296)	Q3 (3.026–3.358) (n = 292)	Q4 (3.358–3.828) (n = 294)	Q5 (3.828–9.533) (n = 295)
Mean age (95% CI) (yr)	59.6 (58.5–60.7)	52.9 (50.4–55.3)	58.25 (56.1–60.4)	60.71 (58.4–63.0)	64.85 (62.9–66.8)	64.65 (62.6–66.7)
Sex						
Male	717 (43.7)	167 (48.6)	155 (49.9)	133 (39.7)	144 (41.6)	118 (35.8)
Female	755 (56.3)	128 (51.4)	141 (50.1)	159 (60.3)	150 (58.4)	177 (64.2)
Race and ethnicity						
Mexican American	93 (2.5)	23 (2.5)	26 (3.4)	13 (2.4)	11 (1.3)	20 (2.5)
Other Hispanic	83 (2.2)	22 (2.6)	14 (1.9)	21 (3.0)	16 (2.3)	10 (1.3)
Non-Hispanic White	1035 (87.3)	205 (87.9)	212 (89.1)	205 (86.7)	215 (88.5)	198 (83.3)
Non-Hispanic Black	207 (5.3)	30 (3.0)	33 (4.1)	42 (6.0)	42 (5.4)	60 (9.0)
Other†	54 (2.8)	15 (4.0)	11 (1.6)	11 (1.9)	10 (2.5)	7 (4.0)
Family poverty income ratio						
≤1.3	336 (13.9)	62 (11.9)	73 (15.8)	67 (14.6)	61 (12.4)	73 (15.0)
>1.3 to 3.5	533 (31.6)	105 (29.7)	94 (24.9)	116 (38.3)	110 (31.5)	108 (35.3)
>3.5	477 (46.1)	103 (51.5)	107 (50.4)	77 (35.8)	98 (48.6)	92 (41.8)
Educational attainment						
Less than high school	377 (16.9)	64 (12.7)	62 (13.0)	91 (25.3)	76 (18.2)	84 (17.0)
High school or equivalent	337 (21.1)	77 (22.0)	64 (20.2)	70 (24.0)	52 (14.3)	74 (25.2)
College or above	758 (61.9)	154 (65.3)	170 (66.7)	131 (50.8)	166 (67.5)	137 (57.8)
Marital status						
Married	864 (63.8)	178 (62.6)	180 (69.4)	167 (64.2)	176 (66.4)	163 (55.3)
Never married	90 (5.1)	26 (7.7)	22 (6.6)	18 (3.4)	13 (3.0)	11 (3.9)
Living with partner	50 (3.9)	16 (5.3)	12 (3.8)	15 (6.5)	3 (1.1)	4 (2.6)
Other‡	467 (27.1)	75 (24.4)	82 (20.2)	92 (25.9)	101 (29.5)	117 (38.1)
Mean BMI (95% CI) (kg/m^2^)	28.1 (27.7–28.5)	26.4 (25.7–27.2)	27.5 (26.8–28.3)	28.4 (27.5–29.2)	28.9 (28.0–29.8)	30.1 (29.0–31.2)
Smoking status						
Never	663 (47.0)	125 (41.4)	137 (51.2)	128 (42.3)	140 (54.0)	133 (46.9)
Former	581 (37.7)	101 (34.1)	113 (34.2)	119 (41.4)	124 (39.3)	124 (41.2)
Current	227 (15.2)	69 (24.4)	46 (14.6)	45 (16.2)	29 (6.5)	38 (11.9)
CVD	361 (18.8)	52 (12.8)	64 (13.8)	67 (17.9)	71 (20.5)	107 (32.5)
Hypertension	837 (50.7)	141 (39.9)	145 (41.4)	182 (57.4)	169 (55.7)	200 (64.7)
Diabetes	281 (15.1)	56 (14.4)	54 (15.1)	57 (14.5)	49 (14.0)	65 (18.1)
Respiratory symptoms	716 (45.6)	126 (39.9)	130 (37.9)	145 (50.9)	152 (47.6)	163 (55.2)
Chronic lung disease	343 (23.6)	65 (23.8)	65 (18.1)	71 (25.2)	72 (26.5)	70 (25.5)

BMI = body mass index, CI = confidence interval, CVD = cardiovascular disease, LAR = lactate dehydrogenase to serum albumin ratio, NHANES = National Health and Nutrition Examination Survey.

* Values are numbers (percentages) unless stated otherwise. Weighted to be nationally representative. The sum of weighted percentages may not equal 100% due to missing data.

† Other includes any other race or ethnicity other than Mexican American, other Hispanic, non-Hispanic White, or non-Hispanic Black.

‡ Including widowed, divorced, or separated individuals.

### 3.2. LAR and mortality in cancer

**Figure 2.**
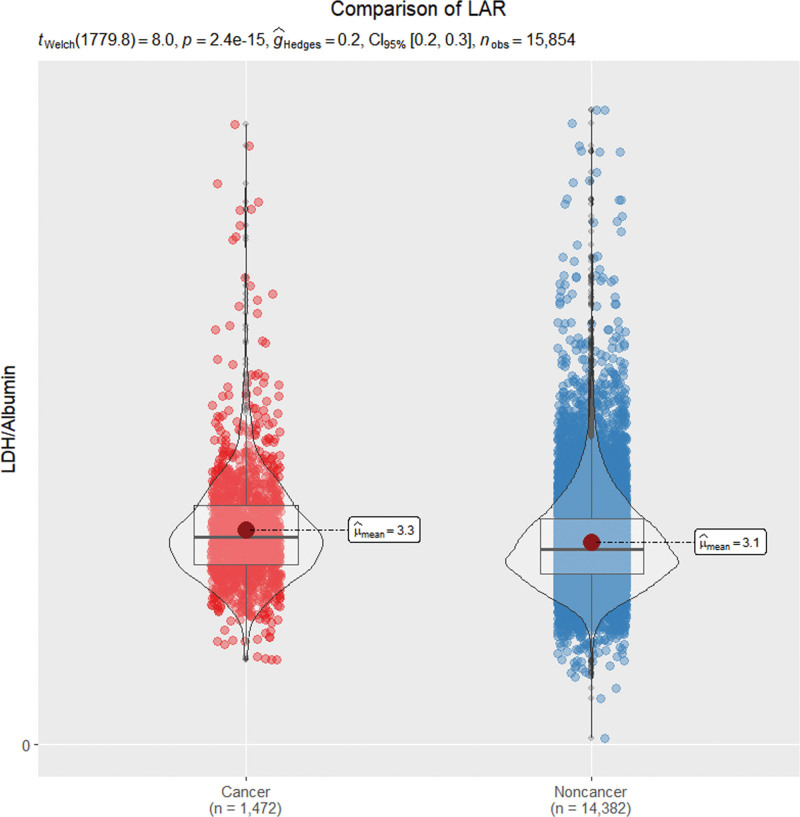
Comparison of LAR between cancer patients and health. LAR is significantly higher in patients with cancer, compared with patients without cancer. Statistical analysis is performed by *t*-test, *P* < .001. CI = confidence interval, LAR = lactate-dehydrogenase-to-albumin ratio, LDH = lactate dehydrogenase.

**Figure 3.**
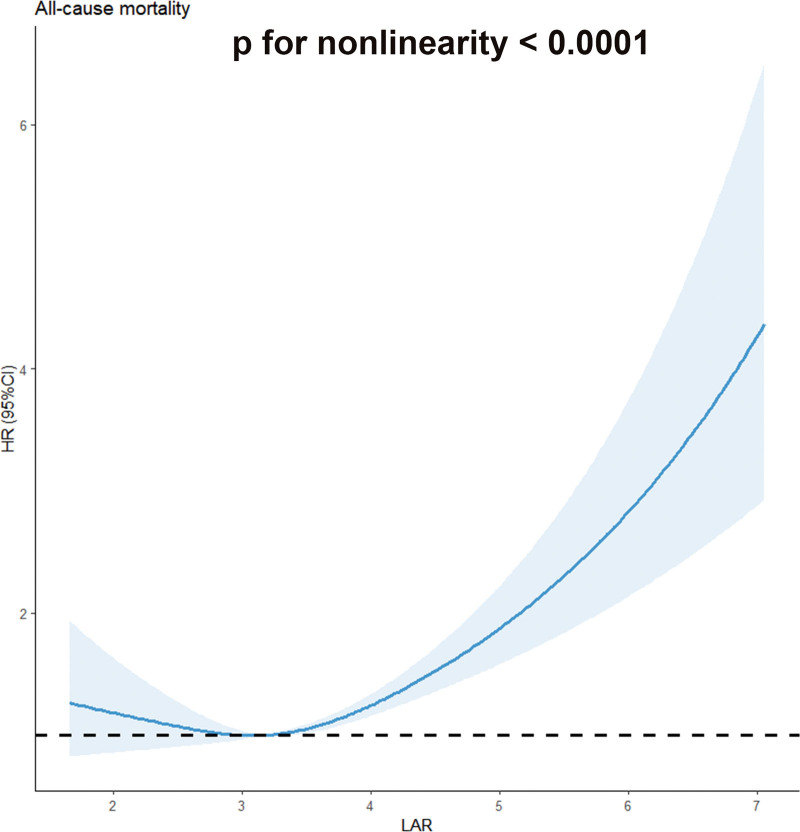
Kaplan–Meier survival estimates between LAR with all-cause mortality. High LAR is associated with higher risk of all-cause death, confirmed by Cox proportional hazards regression models, *P* < .0001. CI = confidence interval, HR = hazard ratio, LAR = lactate-dehydrogenase-to-albumin ratio.

As can be seen in Figure 2, the mean LAR level was significantly higher in the cancer group than in the non-cancer group (*P* < .001). More importantly, higher LAR quintiles are linked to increased mortality risk, with the highest quintile (Q5) showing a 43% higher risk (HR = 1.43, 95% CI: 1.05–1.95) compared to the reference group (Table 2). Furthermore, by smooth curve fitting, the nonlinear relationship between LAR levels and all-cause mortality was assessed after adjusting for multiple potential confounders. The restricted cubic spline analysis revealed a significant nonlinear association between LAR levels and all-cause mortality in cancer patients, with higher LAR levels being linked to a higher mortality risk (*P* for nonlinearity < .0001, shown in Fig. 3).

**Table 2:** Association of LAR with all-cause mortality among US cancer survivors.

Variable	Non-adjusted model	*P* value	Model I*	*P* value	Model II†	*P* value
Per 1 point increase in LAR	1.48 (1.36–1.61)	<.001	1.35 (1.22–1.48)	<.001	1.33 (1.21–1.47)	<.001
Q1 LAR 0–2.681	1 (Reference)		1 (Reference)		1 (Reference)	
Q2 LAR 2.681–3.026	1.06 (0.78–1.45)	.70	1.03 (0.74–1.43)	.86	1.01 (0.73–1.40)	.95
Q3 LAR 3.026–3.358	1.07 (0.79–1.46)	.67	0.87 (0.62–1.21)	.41	0.84 (0.60–1.17)	.30
Q4 LAR 3.358–3.828	1.27 (0.95–1.71)	.11	0.89 (0.65–1.23)	.48	0.87 (0.63–1.21)	.41
Q5 LAR 3.828–9.533	2.07 (1.57–2.73)	<.001	1.49 (1.10–2.02)	.01	1.43 (1.05–1.95)	.02

BMI = body mass index, LAR = lactate dehydrogenase to serum albumin ratio.

* Analyses were adjusted for age, sex (male/female), race and ethnicity (Mexican American, other Hispanic, non-Hispanic White, non-Hispanic Black, other [including American Indian/Alaska Native/Pacific Islander, Asian, multiracial]), family poverty income ratio, educational attainment (<high school graduate, high school graduate or general equivalency diploma, ≥some college), marital status (married, never married, living with partner, other [including widowed, divorced, separated individuals]), BMI, smoking status (never, former, current), and National Health and Nutrition Examination Survey cycles (2007–2008, 2009–2010, 2011–2012).

† Fully adjusted model, further adjusted for cardiovascular disease (yes/no), hypertension (yes/no), diabetes (yes/no) respiratory symptoms (yes/no), and chronic lung disease (yes/no).

### 3.3. LAR and respiratory disease mortality in cancer

**Figure 4.**
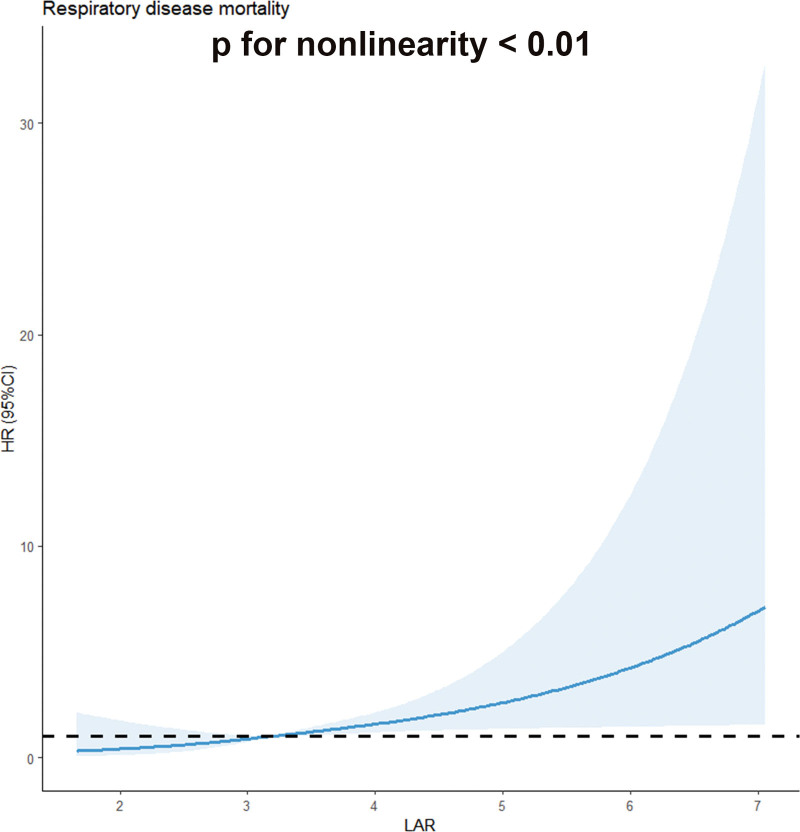
Kaplan–Meier survival estimates between LAR with respiratory disease mortality. High LAR is associated with higher risk of respiratory disease death, confirmed by Cox proportional hazards regression models, *P* < .01. CI = confidence interval, HR = hazard ratio, LAR = lactate-dehydrogenase-to-albumin ratio.

As shown in Table 3 and Figure 4, LAR levels were significantly associated with an increased respiratory disease mortality in cancer survivors (*P* for nonlinearity < .01). In the fully adjusted model, a per 1 point increase in LAR was associated with a 72% higher risk of respiratory disease mortality (HR = 1.72, 95% CI: 1.23–2.39). Specifically, participants in the highest LAR quintile exhibited a substantially higher risk (HR = 4.87, 95% CI: 1.25–18.9) compared to the reference group. These findings underscore the potential of LAR as a prognostic indicator for respiratory disease mortality in cancer patients. Moreover, sensitivity analysis was conducted to sequentially exclude statistical methods, age, sex, and follow-up duration from the model. The effect estimates for the primary exposure remained virtually unchanged (see Table 4), indicating that the conclusions are independent of these demographic and temporal factors.

**Table 3:** Association of LAR with respiratory disease mortality among US cancer survivors.

Variable	Non-adjusted model	*P* value	Model I*	*P* value	Model II†	*P* value
Per 1 point increase in LAR	1.57 (1.18–2.09)	.002	1.61 (1.16–2.24)	.008	1.72 (1.23–2.39)	.001
Q1 LAR 0–2.681	1 (Reference)		1 (Reference)		1 (Reference)	
Q2 LAR 2.681–3.026	1.48 (0.41–5.25)	.54	2.34 (0.57–9.56)	.48	2.24 (0.53–9.40)	.27
Q3 LAR 3.026–3.358	1.73 (0.51–5.92)	.38	1.40 (0.32–6.07)	.54	1.51 (0.34–6.79)	.59
Q4 LAR 3.358–3.828	2.00 (0.60–6.65)	.26	2.40 (0.62–9.31)	.34	2.41 (0.61–9.57)	.21
Q5 LAR 3.828–9.533	3.73 (1.22–11.4)	.02	4.04 (1.07–15.2)	.04	4.87 (1.25–18.9)	.02

BMI = body mass index, LAR = lactate dehydrogenase to serum albumin ratio.

* Analyses were adjusted for age, sex (male/female), race and ethnicity (Mexican American, other Hispanic, non-Hispanic White, non-Hispanic Black, other [including American Indian/Alaska Native/Pacific Islander, Asian, multiracial]), family poverty income ratio, educational attainment (<high school graduate, high school graduate or general equivalency diploma, ≥some college), marital status (married, never married, living with partner, other [including widowed, divorced, separated individuals]), BMI, smoking status (never, former, current), and National Health and Nutrition Examination Survey cycles (2007–2008, 2009–2010, 2011–2012).

† Fully adjusted model, further adjusted for cardiovascular disease (yes/no), hypertension (yes/no), diabetes (yes/no) respiratory symptoms (yes/no), and chronic lung disease (yes/no).

**Table 4:** Associations of LAR with all-cause and respiratory disease mortality among US cancer survivors: sensitivity analyses.*

Analyses	Per 1 point increase in LAR	
	All causes	Respiratory disease mortality
Main analysis		
HR (95% CI) of model I	1.35 (1.22–1.48)	1.61 (1.16–2.24)
HR (95% CI) of model II	1.33 (1.21–1.47)	1.72 (1.23–2.39)
Multiple imputation		
HR (95% CI) of model I	1.35 (1.23–1.48)	1.55 (1.12–2.13)
HR (95% CI) of model II	1.35 (1.23–1.48)	1.65 (1.19–2.29)
Among individuals aged 40 yr or older		
HR (95% CI) of model I	1.35 (1.22–1.48)	1.62 (1.16–2.24)
HR (95% CI) of model II	1.34 (1.21–1.48)	1.72 (1.23–2.40)
Excluding non-Hispanic Black participants		
HR (95% CI) of model I	1.27 (1.14–1.41)	1.57 (1.10–2.25)
HR (95% CI) of model II	1.26 (1.12–1.41)	1.67 (1.16–2.40)
Excluding deaths that occurred within the first 2 yr of follow-up		
HR (95% CI) of model I	1.23 (1.09–1.38)	1.78 (1.32–2.42)
HR (95% CI) of model II	1.23 (1.09–1.38)	1.89 (1.39–2.59)

BMI = body mass index, CI = confidence interval, HR= hazard ratio, LAR = lactate dehydrogenase to serum albumin ratio.

* Model I included age, sex (male/female), race and ethnicity (Mexican American, other Hispanic, non-Hispanic White, non-Hispanic Black, other [including American Indian/Alaska Native/Pacific Islander, Asian, multiracial]), family poverty income ratio, educational attainment (<high school graduate, high school graduate or general equivalency diploma, ≥some college), marital status (married, never married, living with partner, other [including widowed, divorced, separated individuals]), BMI, smoking status (never, former, current), and National Health and Nutrition Examination Survey cycles (2007–2008, 2009–2010, 2011–2012). Model II additionally included cardiovascular disease (yes/no), hypertension (yes/no), diabetes (yes/no) respiratory symptoms (yes/no), and chronic lung disease (yes/no).

## 4. Discussion

The lactate dehydrogenase to serum albumin ratio (LAR) has emerged as a promising biomarker in the diagnosis and prognosis of various diseases, particularly in tumors.^[5,6,15-18]^ In tumor research, LAR has shown significant prognostic value. Studies have found that increased LAR is associated with poor prognosis in patients with colorectal cancer, bladder cancer, and gastric cancer.^[8,9,19]^

Biochemically, an elevated LAR suggests 2 simultaneous derangements: rising LDH and falling albumin. Previous studies have documented that serum LDH is frequently elevated in cancer patients and correlates with poorer prognosis,^[20,21]^ reflecting tumor progression and increased glycolytic activity. LDH, a key enzyme in anaerobic glycolysis, converting pyruvate to lactate, is often elevated in cancer patients due to the high metabolic activity of tumor cells.^[18,22]^ Interestingly, the LDH has been consistently shown by multiple studies to be elevated across various malignancies and to exert tumor-promoting effects.^[21]^ Notably, the end product of LDH, lactate has also been identified as an oncometabolite that actively fuels tumor progression. Lactate serves as not only the alternative resource for cancer growth but also the signal for the regulation of genes expression.^[23]^ Serum albumin, as a marker of nutritional status and immune function, is often decreased in patients with advanced cancer and chronic diseases.^[24,25]^ Therefore, LAR comprehensively reflects the patient’s metabolic state, nutritional status, and immune function, providing important clues for disease diagnosis and prognosis.

In pulmonary diseases, LAR also has important diagnostic and prognostic significance. For example, increased LAR is associated with poor prognosis in patients with lower respiratory tract infections and pulmonary embolism.^[5,26,27]^ Pulmonary embolism and pulmonary infection can lead to significant inflammation and tissue damage in the lungs, resulting in increased LDH levels and decreased serum albumin levels, thus increasing LAR. Many researches have reported that lactate could hinder the immune system to fight against infection.^[28]^ Specifically, lactate could suppress the RLR signaling by targeting MAVS, in turn heightening type I IFN production to protect mice from viral infection.^[28]^ From there, the increase of the LDH in serum might related to the suppression of the immune system, leading to worsen prognosis. Collectively, an elevated LAR might demonstrate accelerated tumor aggressiveness and malnutrition and immune dysregulation. These processes are mutually reinforcing, generating a vicious cycle that erodes pulmonary reserve and precipitates terminal respiratory disease mortality.

This study further clarifies the association between LAR and mortality in cancer patients, highlighting its potential as a prognostic indicator. Our results show that higher LAR levels are positively correlated with increased all-cause mortality and respiratory disease mortality in cancer patients. Specifically, participants in the highest LAR quintile had a significantly higher risk of all-cause mortality and respiratory disease mortality compared to the reference group. This suggests that LAR can effectively identify cancer patients at high risk of death, providing important references for clinical treatment and prognosis assessment. Additionally, a high LAR may alert clinicians to initiate early respiratory interventions, thereby delaying or preventing the onset of respiratory failure.

Despite the significant findings of this study, some limitations should be acknowledged. First, the study is based on data from the NHANES database, which is an observational study.^[29]^ Therefore, causal relationships cannot be established, and residual confounding factors may exist. Second, the study included a relatively small number of cancer patients, which may limit the statistical power of the results. Third, the study did not collect detailed information on the specific types and stages of cancer and different treatment, which may affect the interpretation of the results. Fourth, due to the incomplete data from NHANES, we cannot rule out that deaths attributed to respiratory causes were accompanied by other events, nor can we exclude the possibility that LAR similarly predicts cardiac or other non-respiratory mortality in cancer patients. From there, large-scale prospective cohort studies should be conducted to further confirm the association between LAR and mortality in cancer patients and to explore the underlying mechanisms.

In conclusion, this study demonstrates that LAR is a significant predictor of respiratory disease mortality in cancer patients. Future research should further explore the mechanisms underlying the association between LAR and mortality and investigate the potential of LAR as a target for clinical interventions.

## Author contributions

**Conceptualization:** Liangjun Tang, Haibo Tan.

**Data curation:** Jide Chen, Haibo Tan.

**Formal analysis:** Liangjun Tang, Jide Chen, Haibo Tan.

**Investigation:** Liangjun Tang.

**Methodology:** Jide Chen.

**Project administration:** Haibo Tan.

**Resources:** Liangjun Tang, Haibo Tan.

**Software:** Haibo Tan.

**Supervision:** Haibo Tan.

**Writing – original draft:** Liangjun Tang.

**Writing – review & editing:** Liangjun Tang.
